# *COBL*, *MKX* and *MYOC* Are Potential Regulators of Brown Adipose Tissue Development Associated with Obesity-Related Metabolic Dysfunction in Children

**DOI:** 10.3390/ijms24043085

**Published:** 2023-02-04

**Authors:** Sarah Abdul Majeed, Helene Dunzendorfer, Juliane Weiner, John T. Heiker, Wieland Kiess, Antje Körner, Kathrin Landgraf

**Affiliations:** 1 Center for Pediatric Research Leipzig (CPL), Hospital for Children & Adolescents, University of Leipzig, 04103 Leipzig, Germany; 2 Medical Department III—Endocrinology, Nephrology, Rheumatology, University of Leipzig Medical Center, 04103 Leipzig, Germany; 3 Helmholtz Institute for Metabolic, Obesity and Vascular Research (HI-MAG) of the Helmholtz Zentrum München at the University of Leipzig and University Hospital Leipzig, 04103 Leipzig, Germany

**Keywords:** adipose tissue, white adipose tissue, brown adipose tissue, adipocyte differentiation, children, obesity, metabolic disease, COBL, MKX, MYOC

## Abstract

Obesity is already accompanied by adipose tissue (AT) dysfunction and metabolic disease in children and increases the risk of premature death. Due to its energy-dissipating function, brown AT (BAT) has been discussed as being protective against obesity and related metabolic dysfunction. To analyze the molecular processes associated with BAT development, we investigated genome-wide expression profiles in brown and white subcutaneous and perirenal AT samples of children. We identified 39 upregulated and 26 downregulated genes in uncoupling protein 1 (UCP1)-positive compared to UCP1-negative AT samples. We prioritized for genes that had not been characterized regarding a role in BAT biology before and selected cordon-bleu WH2 repeat protein (*COBL*), mohawk homeobox (*MKX*) and myocilin (*MYOC*) for further functional characterization. The siRNA-mediated knockdown of *Cobl* and *Mkx* during brown adipocyte differentiation in vitro resulted in decreased *Ucp1* expression, while the inhibition of *Myoc* led to increased *Ucp1* expression. Furthermore, *COBL*, *MKX* and *MYOC* expression in the subcutaneous AT of children is related to obesity and parameters of AT dysfunction and metabolic disease, such as adipocyte size, leptin levels and HOMA-IR. In conclusion, we identify *COBL*, *MKX* and *MYOC* as potential regulators of BAT development and show an association of these genes with early metabolic dysfunction in children.

## 1. Introduction

Obesity manifests early in life and increases the risk of developing type 2 diabetes, cardiovascular diseases, hypertension and metabolic syndrome, and is therefore a risk factor for early death [1]. Obesity develops if the energy intake exceeds the total energy expenditure and eventually this excessive energy is stored in form of lipids in white adipose tissue (WAT) [2,3]. WAT consists of unilocular white adipocytes that have a variable amount of mitochondria, and plays an important role in the endocrine system [4]. Brown adipocytes in contrast have a multilocular structure, are abundant in mitochondria and dissipate energy by the production of heat by non-shivering thermogenesis through the action of uncoupling protein 1 (UCP1) [5]. It was long considered that brown adipose tissue (BAT) was only present in small mammals, infants and young children, but recent studies have detected active BAT in adults though mass and activity decrease with age [6,7]. In humans, BAT depots are mainly confined to the interscapular and supraclavicular region and to lesser extent to the perirenal region in the abdomen (reviewed in [8]). During recent years, it has been shown that an increase in BAT mass can originate either from the maturation of brown adipocyte precursor cells (classical differentiation) or from the transdifferentiation of white adipocytes into brown-like or so-called beige adipocytes, which resemble the characteristic features of brown adipocytes (*UCP1* expression, multilocular lipid droplets, thermogenic activity) and are specifically found in WAT [9,10,11]. The so-called white adipocyte browning can be induced by a variety of factors in vitro and/or in vivo, including cold exposure, β-adrenergic receptor agonists, endocrine factors, e.g., bone morphogenetic protein 7 (BMP7), or pharmacologically active compounds, e.g., rosiglitazone [11,12,13,14]. Based on increasing evidence that BAT activity inversely correlates with measures of obesity and is associated with a beneficial effect on metabolic parameters in children and adults [6,15,16,17,18,19,20], a better understanding of the regulation of BAT development and its age and obesity-associated decrease might lead to new therapeutic strategies to prevent and cure obesity and related comorbidities [4,21,22,23]. We have previously shown the presence of brown-like *UCP1*-expressing adipocytes in WAT samples of different depots, including perirenal and subcutaneous locations, in children of our Leipzig Adipose Tissue (AT) Childhood cohort [24], which was related to age and obesity state [25]. Here, we aimed to identify novel potential regulators of BAT development in children. For this, we investigated genome-wide expression profiles in UCP1-positive and UCP1-negative AT samples from children and characterized the relevance of identified candidate gene expression for brown adipocyte formation in vitro and for childhood obesity and related parameters in vivo.

## 2. Results

### 2.1. Identification of the Candidate Genes Potentially Relevant for BAT Development

In order to identify genes with a potential relevance for BAT development and brown adipocyte formation, we analyzed the Illumina transcriptome profiles of brown (positive for UCP1 protein in histological analyses, UCP1^+^) vs. white (negative for UCP1 protein in histological analyses, UCP1^−^) AT samples of patients included in the Leipzig AT Childhood cohort [24,25,26]. There were six perirenal and four subcutaneous UCP1^+^ AT samples available, and we compared them to two available perirenal and four subcutaneous (matched for age, sex and BMI SDS of probands) UCP1^−^ AT samples. Patient characteristics are shown in Table S2.

**Figure 1:**
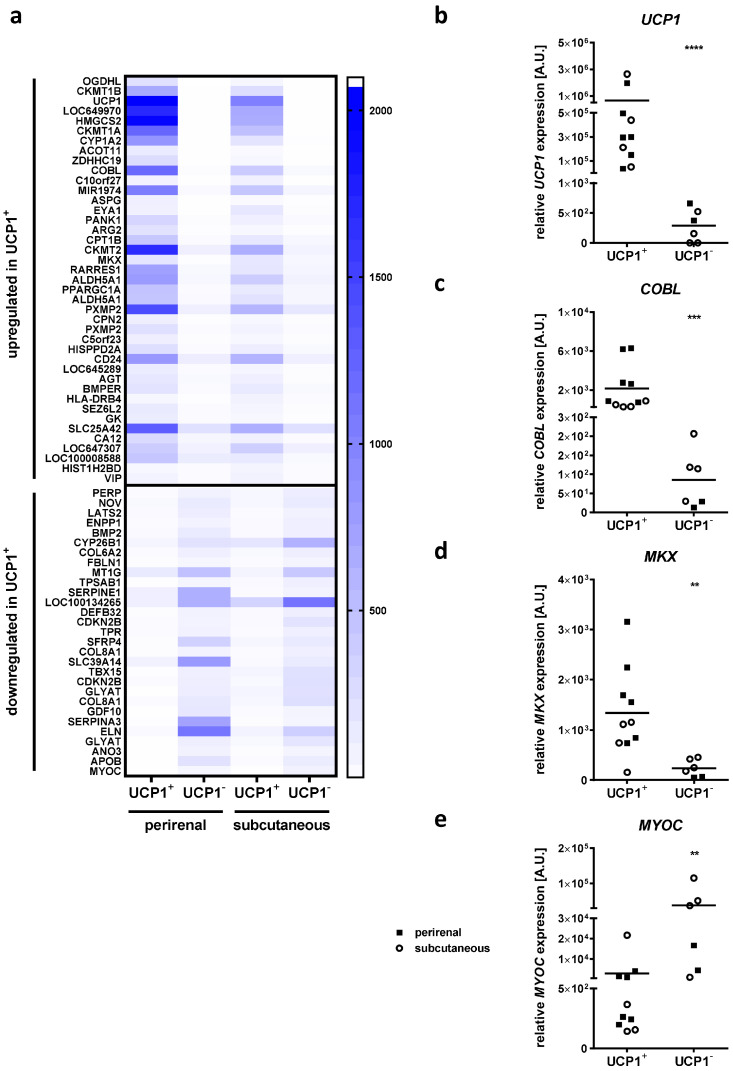
Identification of *COBL*, *MKX* and *MYOC* as regulated genes in brown vs. white children’s adipose tissue (AT) samples. (**a**) Heat map of transcriptome-based expression levels of up- and downregulated genes in perirenal and subcutaneous AT samples positive for the brown adipocyte marker uncoupling protein 1 (UCP1) in histological analyses (UCP1^+^) compared to histologically negative (UCP1^−^) AT samples. Mean over all analyzed samples per group is shown. Expression of (**b**) *UCP1* as well as the candidate genes (**c**) cordon-bleu WH2 repeat protein (*COBL*), (**d**) mohawk homeobox (*MKX*) and (**e**) myocilin (*MYOC*) in UCP1^+^ AT samples was compared to that of UCP1^−^ AT samples using quantitative real-time PCR. Six available perirenal UCP1^+^ AT samples were compared to the 2 available perirenal UCP1^−^ samples. Four available subcutaneous UCP1^+^ AT samples were compared to 4 subcutaneous UCP1^−^ samples matched for similar age, sex and BMI SDS of probands. Scatter dot plots show individual samples grouped into perirenal (filled square) and subcutaneous (open circle) AT samples. The grand mean over all samples is indicated by a horizontal line. Statistical analyses were performed using Student’s t-test and significant *p*-values are indicated. ** *p* < 0.01; *** *p* < 0.001; **** *p* < 0.0001.

To identify potential regulators of brown adipocyte differentiation and/or function, we determined differentially expressed genes in each of the depots by comparing UCP1^+^ to UCP1^−^ AT samples and applying a threshold of a least 2.5-fold up or down regulation of gene expression. By doing so, we identified 2229 differentially expressed probes in the perirenal AT (1395 upregulated and 834 downregulated) and 394 differentially expressed probes in the subcutaneous AT (249 upregulated and 145 downregulated). We further narrowed down potential candidate regulators of BAT development by merging the candidate gene lists of both depots, resulting in 70 differentially expressed probes (41 upregulated and 29 downregulated; Figure 1a, Table S3), corresponding to 65 genes (39 upregulated and 26 downregulated; Figure 1a, Table S3).

While the upregulated genes included many genes expressed in mitochondria (e.g., *OGDHL*, *CKMT1A*, *CKMT1B*, *UCP1*, *HMGCS2*, *CYP1A2*), many of the downregulated genes were related to extracellular structure organization (e.g., *COL6A2*, *COL8A1*, *ELN*, *FBLN1*, *TPSAB1*). We screened the candidate gene list for genes that had not previously been described in the context of mitochondria and/or BAT function and had been related to developmental and differentiation processes before. We chose *COBL* (cordon-bleu WH2 repeat protein; 29.5-fold increase in UCP1^+^ compared to UCP1^−^ AT samples) and *MKX* (mohawk homeobox; 8.7-fold increase in UCP1^+^ compared to UCP1^−^ AT samples) as upregulated genes fulfilling these criteria, and *MYOC* (myocilin; decreased to 4% in UCP1^+^ compared to UCP1^−^ AT samples) as the top downregulated gene for further in vitro studies. As an actin nucleator, *COBL* has been shown to play a role in neuronal morphological and developmental processes [27,28], and the overexpression of *COBL* in BAT compared to WAT has previously been shown in independent studies supporting the hypothesis that it might be involved in BAT-specific processes [29,30]. *MKX* has been described as a transcription factor involved in cellular differentiation processes [31,32], in particular the differentiation of bone-marrow-derived mesenchymal stem cells in the context of limb development [33], making it a potential candidate regulator of processes related to brown adipocyte differentiation. *MYOC* is known as a glaucoma-associated protein and is produced in mesenchymal stem cells, stimulating their osteogenic differentiation [34,35].

We confirmed the results from transcriptome analyses using quantitative real-time PCR showing a significant upregulation of *UCP1* (2300-fold, *p* < 0.0001; Figure 1b), *COBL* (25.2-fold, *p* = 0.0001; Figure 1c) and *MKX* (5.6-fold, *p* = 0.0012; Figure 1d), and a significant downregulation of *MYOC* (decrease to 8%, *p* = 0.0025; Figure 1e) expression in UCP1^+^ compared to UCP1^−^ AT samples.

### 2.2. Effect of Cold Exposure on Candidate Gene Expression in AT Depots of Mice

**Figure 2:**
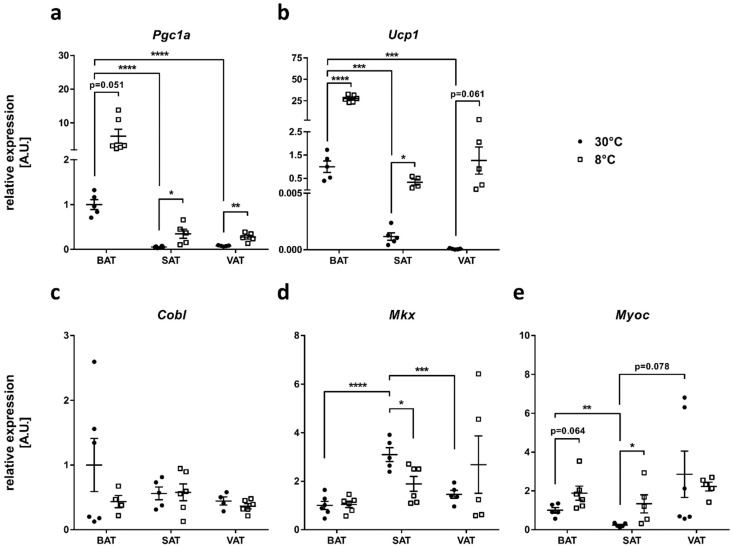
Effect of cold exposure on expression of the candidate genes *Cobl*, *Mkx* and *Myoc* in different adipose tissue (AT) depots of mice. Relative expression of the brown adipocyte marker genes (**a**) peroxisome proliferator-activated receptor gamma coactivator 1-alpha (*Pgc1a*) and (**b**) uncoupling protein 1 (*Ucp1*) as well as the candidate genes (**c**) cordon-bleu WH2 repeat protein (*Cobl*), (**d**) mohawk homeobox (*Mkx*) and (**e**) myocilin (*Myoc*) in brown AT (BAT), subcutaneous AT (SAT, inguinal white AT (WAT)) and visceral AT (VAT, epigonadal WAT) was compared between mice either kept at thermoneutrality (30 °C) or subjected to cold exposure (8 °C) for a period of 7 days. Data of four to six mice per group are presented as fold change compared to BAT expression levels at 30 °C and are given as mean ± SEM. Statistical analyses were performed using Student’s t-test and significant *p*-values are indicated. * *p* < 0.05; ** *p* < 0.01; *** *p* < 0.001; **** *p* < 0.0001.

To assess whether the candidate genes *COBL*, *MYOC* and *MKX* might be involved in processes related to AT browning, we investigated the effect of cold exposure on candidate gene expression in different fat depots of mice. At thermoneutrality, BAT showed the highest expression of the BAT marker genes *Pgc1a* (approx. 18 times higher compared to subcutaneous AT (SAT, inguinal WAT) and 12 times higher compared to visceral AT (VAT, epigonadal WAT); Figure 2a) and *Ucp1* (approx. 850 times higher compared to SAT and 12,000 times higher compared to VAT; Figure 2b). As expected, the exposure of mice to a cold temperature for a period of 7 days led to an increase in both *Pgc1a* (Figure 2a) and *Ucp1* (Figure 2b) expression in all fat depots analyzed.

We then measured the expression of the candidate genes *Cobl*, *Mkx* and *Myoc* in the three fat depots and compared their expression between the mice kept at thermoneutrality and those exposed to cold (Figure 2c–e). At thermoneutrality, *Cobl* expression was not significantly different between the depots, and cold exposure did not affect *Cobl* expression in BAT, SAT or VAT (Figure 2c). Under thermoneutral conditions, *Mkx* showed the highest expression in SAT being increased 3.1 fold compared to BAT and increased 2.1 fold compared to VAT. After cold exposure, *Mkx* expression in SAT significantly decreased to about 60%, while *Mkx* expression in BAT and VAT was not significantly altered (Figure 2d). *Myoc* expression showed no significant differences in the BAT and VAT of mice kept at thermoneutrality (*p* = 0.1948), while expression in SAT was significantly lower (23% of the expression in BAT; 8% of the expression in VAT). *Myoc* expression significantly increased 5.7 fold in SAT and showed a trend towards an increase in BAT (1.9-fold) in cold-exposed mice compared to mice kept at thermoneutrality, while expression in VAT remained unchanged (Figure 2e). In summary, *Mkx* and *Myoc* but not *Cobl* expression is affected by adipocyte browning in the AT depots of mice.

### 2.3. Regulation of Candidate Gene Expression during Brown Adipocyte Differentiation

**Figure 3:**
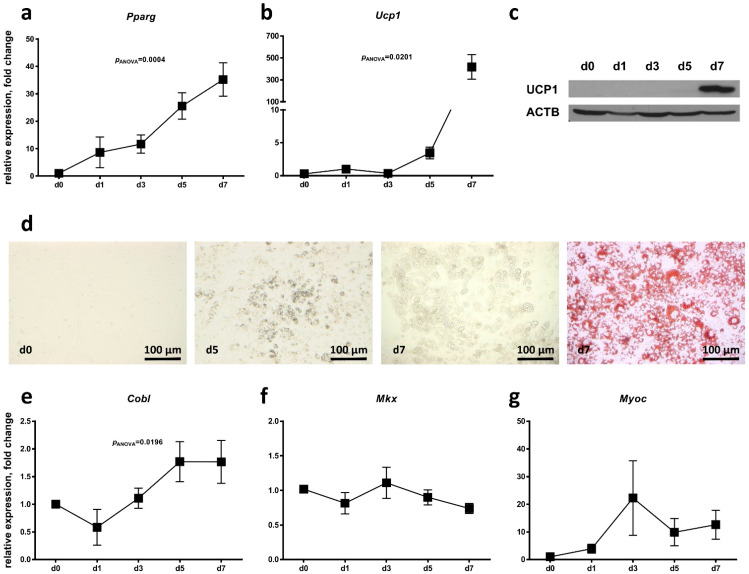
Regulation of candidate gene expression during brown adipocyte differentiation of HIB1B cells. HIB1B cells were subjected to adipocyte differentiation for a period of 7 days. Relative expression of the marker genes (**a**) peroxisome proliferator-activated receptor gamma (*Pparg*) and (**b**) uncoupling protein 1 (*Ucp1*) was measured to verify efficient differentiation into brown adipocytes. (**c**) UCP1 protein levels of one exemplary experiment were analyzed by immunoblot at different time points of brown adipocyte differentiation. Detection of β-Actin (ACTB) served as loading control. (**d**) Microscopic bright-field images of HIB1B cells before adipogenic induction (day 0) and on days 5 and 7 of adipocyte differentiation. Lipid accumulation in differentiated HIB1B cells was visualized by Oil-Red-O staining. Relative expression of the candidate genes (**e**) cordon-bleu WH2 repeat protein (*Cobl*), (**f**) mohawk homeobox (*Mkx*) and (**g**) myocilin (*Myoc*) was analyzed before adipogenic induction (day 0) and at different time points of brown adipocyte differentiation. Data are compared to day 0 and presented as mean ± SEM of five independent experiments each measured in triplicate. Significant *p*-values determined by one-way ANOVA are given.

We next evaluated whether the candidate genes *Cobl*, *Mkx* and *Myoc* are regulated during classical BAT development. Therefore, we used the previously described murine HIB1B brown adipocyte cell culture model [36], and investigated candidate gene expression during in vitro differentiation from brown adipose progenitor cells into brown adipocytes. We confirmed efficient brown adipocyte differentiation over a period of 7 days from significant increases in *Pparg* expression (Figure 3a), *Ucp1* mRNA (Figure 3b) and UCP1 protein levels (Figure 3c) as well as the visualization of lipid accumulation by Oil-Red-O staining on day 7 of brown adipocyte differentiation (Figure 3d). *Cobl* expression was increased about 1.7-fold on day 7 of brown adipocyte differentiation compared to day 0 before adipogenic induction (Figure 3e), while both *Mkx* and *Myoc* expression were not significantly regulated (Figure 3f,g).

### 2.4. Effect of Candidate Gene Knockdown on Brown Adipocyte Differentiation

**Figure 4:**
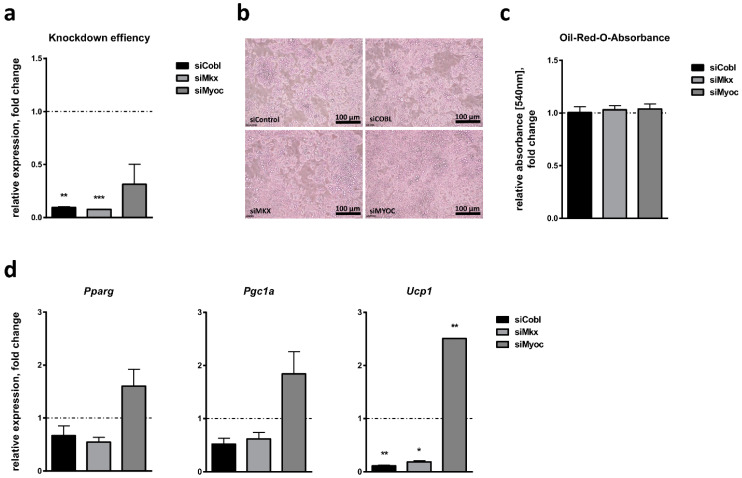
Effect of candidate gene knockdown on brown adipocyte differentiation of HIB1B cells. (**a**) Cordon-bleu WH2 repeat protein (*Cobl*), mohawk homeobox (*Mkx*) and myocilin (*Myoc*) expression was analyzed 48 h after siRNA-mediated candidate gene knockdown and directly before adipogenic induction (day 0). (**b**) Microscopic bright-field images of HIB1B cells treated with the indicated siRNAs on day 7 of brown adipocyte differentiation. (**c**) Lipid accumulation as a measure of adipogenic capacity was quantified by Oil-Red-O staining and absorbance measurement. (**d**) Relative expression of the adipogenic marker genes peroxisome proliferator-activated receptor gamma (*Pparg*), peroxisome proliferator-activated receptor gamma coactivator 1-alpha (*Pgc1a*) and uncoupling protein 1 (*Ucp1*) was measured on day 7 of adipocyte differentiation. Bar plots represent mean ± SEM of the two independent experiments compared to cells treated with control siRNA (dotted line), each measured in triplicate. Significant *p*-values (Student’s *t*-test) are indicated. * *p* < 0.05; **, *p* < 0.01; *** *p* < 0.001.

To investigate the role of *Cobl*, *Mkx* and *Myoc* during classical brown adipocyte differentiation in more detail, we performed siRNA-mediated knockdown analyses in HIB1B cells. The successful downregulation of each candidate was verified 48 h post transfection, which corresponded to day 0 of the differentiation protocol. SiRNA-mediated knockdown led to the reduced expression of the three candidate genes with different efficiencies: *Cobl* expression was reduced to about 9.5%, *Mkx* expression to 7.8% and *Myoc* expression to 31.3%, compared to cells transfected with a control siRNA (Figure 4a). Microscopic pictures on day 7 of brown adipocyte differentiation and Oil-Red-O staining did not show obvious differences in lipid accumulation between cells treated with siRNAs targeting *Cobl*, *Mkx* or *Myoc*, and a non-targeting control siRNA (Figure 4b,c). We next assessed the effect of candidate gene knockdown on the expression of markers of adipocyte differentiation per se (*Pparg*) and brown adipocyte formation in particular (*Pgc1a*, *Ucp1*) on day 7 of adipocyte differentiation. There was no significant effect of candidate gene knockdown on *Pparg* expression (Figure 4d), indicating no effect on adipocyte differentiation in general. Furthermore, candidate gene knockdown did not significantly affect *Pgc1a* expression. However, the inhibition of both *Cobl* and *Mkx* expression was associated with reduced *Ucp1* expression (11% for siCobl and 19% for siMkx compared to control siRNA), while the inhibition of *Myoc* led to significantly increased *Ucp1* expression levels (2.5-fold compared to control; Figure 4d).

### 2.5. Regulation of Candidate Gene Expression during White Adipocyte Browning

We next addressed a potential role of the candidate genes, *Cobl*, *Mkx* and *Myoc*, during white adipocyte browning by investigating gene expression in murine 3T3L1 cells undergoing adipocyte differentiation in presence or absence of the inducers of adipocyte browning, rosiglitazone [37,38,39] or BMP7 [13,39,40].

**Figure 5:**
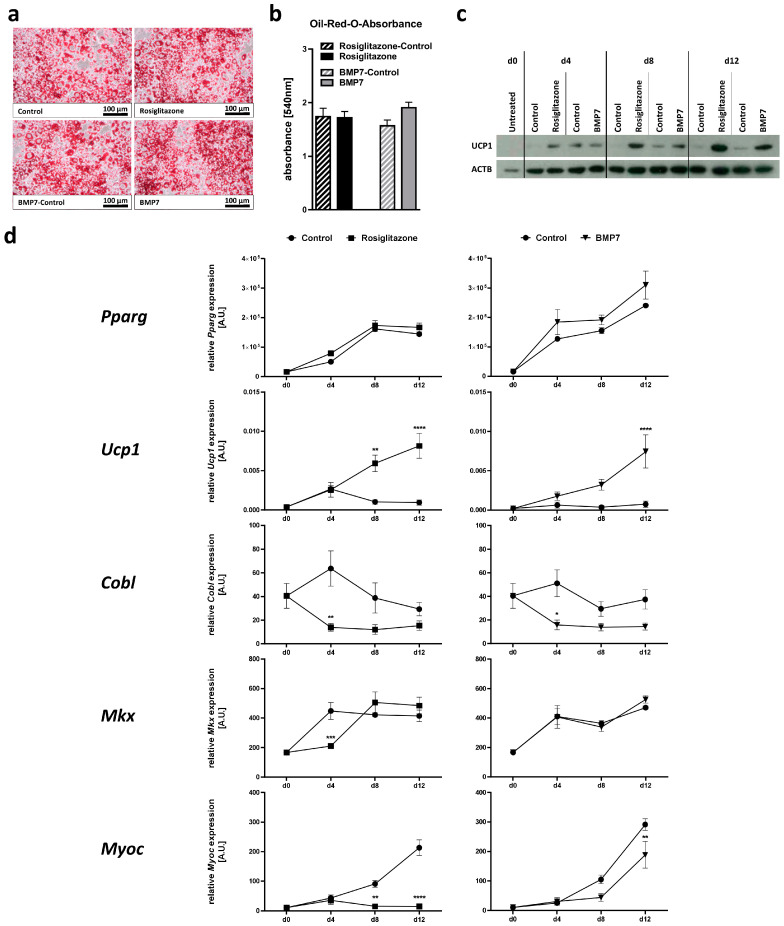
Regulation of candidate gene expression during white adipocyte browning of 3T3-L1 cells. 3T3-L1 cells were subjected to adipocyte differentiation and white adipocyte browning using either rosiglitazone or bone morphogenetic protein 7 (BMP7) for a period of 12 days. Efficient adipocyte differentiation was confirmed by (**a**) Oil-Red-O staining and subsequent (**b**) extraction and absorbance measurement. (**c**) Browning of white adipocytes was verified by immunoblot detection of the brown adipocyte marker uncoupling protein 1 (UCP1) in one exemplary experiment. Detection of β-Actin (ACTB) served as loading control. (**d**) Relative expression of the white and/or brown adipogenic marker genes peroxisome proliferator-activated receptor gamma (*Pparg*) and *Ucp1* as well as the candidate genes cordon-bleu WH2 repeat protein (*Cobl*), mohawk homeobox (*Mkx*) and myocilin (*Myoc*) was analyzed at different time points of adipocyte differentiation. Data are given as mean ± SEM of the three independent experiments each measured in triplicate. Significant *p*-values (determined by two-way ANOVA and Sidak’s multiple comparison test) for differences between treated cells and control cells are indicated. * *p* < 0.05; ** *p* < 0.01; *** *p* < 0.001; **** *p* < 0.0001.

We verified the successful adipocyte differentiation by Oil-Red-O staining of cells on day 12 of differentiation (Figure 5a). Oil-Red-O absorbance as a quantitative measure of lipid accumulation did not show differences between cells treated with rosiglitazone or BMP7 and untreated control cells (Figure 5b). To prove the successful browning of white adipocytes, we performed Western Blot analyses using UCP1-specific antibodies. Both rosiglitazone and BMP7 led to an upregulation of UCP1 protein levels, with the strongest effects detectable from day 8 of differentiation onwards (Figure 5c). In line with this, the expression levels of *Pparg* steadily increased throughout differentiation, from day 0 till day 12 (9-fold for rosiglitazone control, 15-fold for BMP7 control; both *p* < 0.0001), and did not show differences between cells treated with rosiglitazone or BMP7 or untreated cells (Figure 5d). *Ucp1* expression levels were not affected by the differentiation process per se but were significantly increased in cells treated with rosiglitazone or BMP7 compared to control cells from day 8 onwards (rosiglitazone: 5.8-fold on day 8, 8.6-fold on day 12; BMP7: 9.2-fold on day 8, 10-fold on day 12; Figure 5c,d).

During classical white adipocyte differentiation, i.e., in absence of inducers of adipocyte browning, *Cobl* expression was not changed, while both *Mkx* and *Myoc* expression significantly increased from day 0 to day 12 (*Mkx*: 2.5-fold for rosiglitazone control, *p* = 0.0001 and 2.8-fold for BMP7 control, *p* < 0.0001; *Myoc*: 20-fold for rosiglitazone control and 27-fold for BMP7 control, both *p* < 0.0001; Figure 5d). White adipocyte browning induced by rosiglitazone or BMP7 treatment did not consistently affect *Mkx* expression, while there was a significant downregulation of the expression of *Cobl* on day 4 of the white adipocyte browning process (22% of control for rosiglitazone and 31% for BMP7), which was, however, before a detectable increase in *Ucp1* expression (Figure 5d). Interestingly, *Myoc* was significantly downregulated on day 8 and day 12 of white adipocyte browning (7% of control for rosiglitazone and 65% for BMP7 on day 12), which was paralleled by increased *Ucp1* expression levels (Figure 5d).

### 2.6. Effect of Candidate Gene Knockdown on White Adipocyte Browning

**Figure 6:**
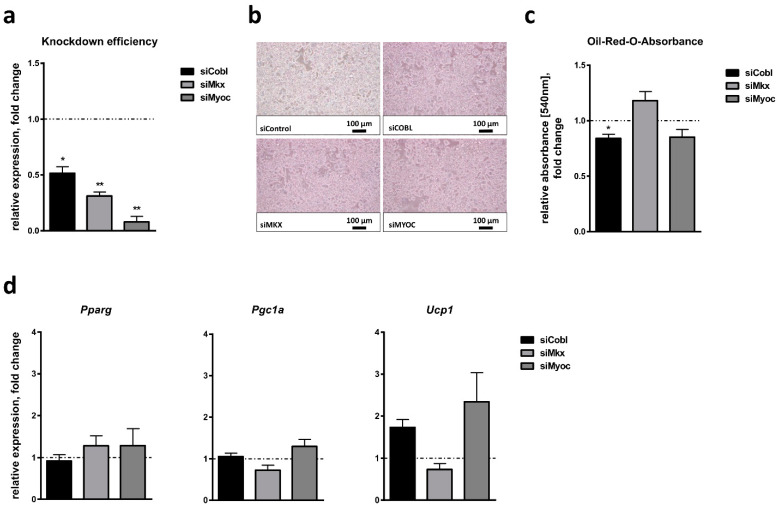
Effect of candidate gene knockdown on white adipocyte browning of 3T3-L1 cells. (**a**) Cordon-bleu WH2 repeat protein (*Cobl*), mohawk homeobox (*Mkx*) and myocilin (*Myoc*) expression was analyzed 48 h after siRNA-mediated candidate gene knockdown and directly before adipogenic induction (day 0). Adipocyte differentiation was performed in the presence of rosiglitazone as an inducer of adipocyte browning. (**b**) Microscopic bright-field images of 3T3-L1 cells treated with the indicated siRNAs on day 8 of adipocyte differentiation. (**c**) Lipid accumulation as a measure of adipogenic capacity was quantified by Oil-Red-O staining and absorbance measurement. (**d**) Relative expression of the white and/or brown adipogenic marker genes peroxisome proliferator-activated receptor gamma (*Pparg*), peroxisome proliferator-activated receptor gamma coactivator 1-alpha (*Pgc1a*) and uncoupling protein 1 (*Ucp1*) was measured on day 8 of adipocyte differentiation. Bar plots show results of the three independent experiments each measured in triplicate. Data are given as mean ± SEM compared to cells treated with control siRNA (dotted line). Significant *p*-values determined by Student’s t-test are indicated. * *p* < 0.05; ** *p* < 0.01.

We next assessed the relevance of *Cobl*, *Mkx* and *Myoc* for the browning of white adipocytes using siRNA-mediated knockdown analyses in 3T3-L1 cells undergoing adipocyte differentiation in the presence of an inducer of adipocyte browning. We decided to restrict the analyses to rosiglitazone only since our previous results showed that both rosiglitazone and BMP7 led to a similar effect on the white adipocyte browning of 3T3-L1 cells and a comparable response of candidate gene expression (Figure 5). Furthermore, we analyzed an effect of candidate gene knockdown after 8 days of white adipocyte browning since this period already showed a significant effect in the previous experiments (Figure 5c,d). Similar to HIB1B cells, siRNA-mediated knockdown in 3T3-L1 cells resulted in the decreased expression of the three candidates to different extents: While *Cobl* expression was downregulated to 53% in cells treated with gene-specific siRNAs compared to non-target control siRNA-treated cells, *Mkx* was inhibited to 31% and *Myoc* to 8% (Figure 6a). The knockdown of candidate genes did not result in a general inhibition of lipid accumulation as shown in the microscopy of differentiated adipocytes (Figure 6b). Measurement of Oil-Red-O absorbance on day 8 of differentiation revealed a significant but only slight decrease in lipid accumulation after treatment with siRNA targeting *Cobl* (84% compared to control siRNA), which was not the case for siRNAs targeting *Mkx* or *Myoc* (Figure 6c). When we assessed the effect of candidate gene knockdown on the expression of *Pparg* as a marker of adipocyte differentiation per se, we did not observe differences between cells treated with siRNAs directed against *Cobl*, *Mkx* or *Myoc* and cells treated with control siRNA (Figure 6d). Similarly, there was no effect on *Pgc1a* expression (Figure 6d). In contrast, while the knockdown of *Mkx* did not affect the expression of the brown adipocyte marker *Ucp1*, the knockdown of *Cobl* and *Myoc* both seemed to be associated with slightly increased *Ucp1* expression levels, though this trend was not statistically significant (1.7-fold for siCobl, *p* = 0.0614; 2.5-fold for siMyoc, *p* = 0.1431; Figure 6d).

### 2.7. Association of Candidate Gene Expression in AT with Childhood Obesity

Finally, we aimed to investigate the relevance of the candidate genes for childhood obesity in more detail. For this we analyzed candidate gene expression in 272 AT samples of children included in the Leipzig AT Childhood cohort and assessed a potential correlation with obesity-related anthropometric and metabolic parameters as well as with parameters of AT biology and function. Cohort characteristics are provided in Table S4.

We first investigated the impact of sex and age on candidate gene expression in the AT of children independent of obesity, by performing analyses in the subgroup of normal weight children. While we did not observe an association of *COBL* expression with any of these parameters, both *MKX* and *MYOC* expression showed an association with sex (*MKX*: 221 ± 24 in females vs. 434 ± 56 in males, *p* = 0.003; *MYOC*: 26,140 ± 2810 in females vs. 19,103 ± 2306 in males, *p* = 0.003) and age (*MKX*: R = 0.223, *p* = 0.003; *MYOC*: R = −0.223, *p* = 0.003). Because of this, sex and age were included as covariates in subsequent correlation analyses.

**Figure 7:**
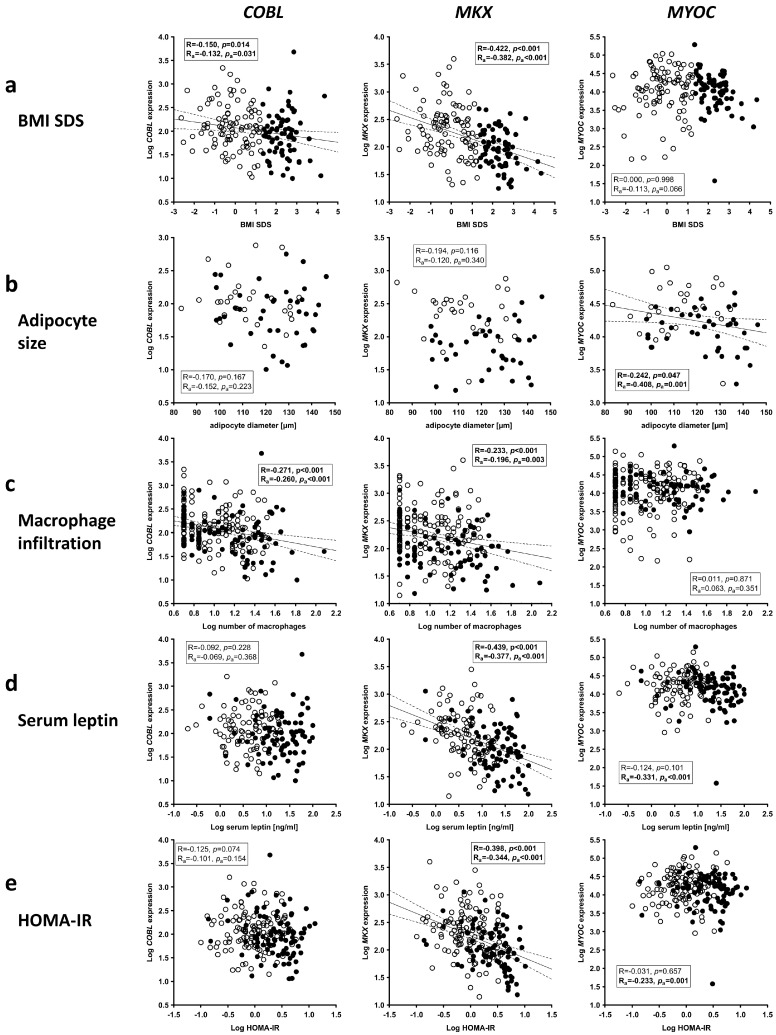
Gene expression of candidate genes in adipose tissue (AT) of children and association with parameters of obesity and AT dysfunction. Cordon-bleu WH2 repeat protein (*COBL*), mohawk homeobox (*MKX*) and myocilin (*MYOC*) expression was analyzed in AT samples of children and an association with (**a**) BMI SDS, (**b**) adipocyte size, (**c**) AT macrophage infiltration, (**d**) serum leptin levels and (**e**) HOMA-IR was assessed. In each scatter plot, Pearson’s correlation coefficient and *p*-value are given two times, unadjusted (R, *p*) and adjusted for age and sex of children (R_a_, *p*_a_). Significant results are indicated in bold. Lean children are represented as open, children with overweight and obesity as closed circles. BMI SDS, body mass index standard deviation score; HOMA-IR, homeostasis model assessment of insulin resistance.

We detected significantly decreased expressions of *COBL* and *MKX* in subcutaneous AT samples of children with overweight and obesity compared to lean children, while the expression of *MYOC* was not altered with obesity (Table S4). In line with this, we observed a significant negative correlation of *COBL* and *MKX* but not *MYOC* expression with the BMI SDS of children (Figure 7a). While *COBL* and *MKX* were not associated with adipocyte size as a measure of adipocyte hypertrophy (Figure 7b), both showed a negative correlation with macrophage infiltration (Figure 7c), which was independent of BMI SDS, as indicated by partial correlation analyses (R_adjusted_ = −0.244, *p*_adjusted_ < 0.001 for *COBL*; R_adjusted_ = −0.148, *p*_adjusted_ = 0.027 for *MKX*). In addition to that, *MKX* but not *COBL* expression showed an inverse and BMI SDS-independent association with serum leptin levels (Figure 7d; R_adjusted_ = −0.168, *p*_adjusted_ = 0.028) and the insulin resistance marker HOMA-IR (Figure 7e; R_adjusted_ = −0.211, *p*_adjusted_ = 0.003).

In contrast, *MYOC* expression showed a negative correlation with adipocyte size as a parameter indicative of adipocyte hypertrophy (Figure 7b), which was independent of the BMI SDS of children (R_adjusted_ = −0.399, *p*_adjusted_ = 0.001), while there was no association with macrophage infiltration into AT as a surrogate marker of AT inflammation (Figure 7c). Furthermore, *MYOC* expression in AT of children was inversely associated with serum leptin levels (Figure 7d) and HOMA-IR (Figure 7e) after adjustment for age and sex of children and these associations were not secondary to the BMI SDS of children (R_adjusted_ = −0.352, *p*_adjusted_ < 0.001 for serum leptin; R_adjusted_ = −0.205, *p*_adjusted_ = 0.003 for HOMA-IR).

In summary, the expression of *COBL*, *MKX* and *MYOC* in AT is related to obesity, AT dysfunction, and the early signs of metabolic disease in children.

## 3. Discussion

Here, we identified *COBL*, *MKX* and *MYOC* as potential regulators of BAT development and function using the genome-wide gene expression profiles of brown and white AT samples from children. We further showed that these genes are involved in classical brown adipocyte differentiation from brown adipose progenitor cells in vitro. Finally, we provide evidence that the expression of *COBL*, *MKX* and *MYOC* in subcutaneous AT is related to obesity, parameters of AT dysfunction, and the early signs of metabolic disease in children.

*COBL* was originally identified in mice as a gene regulating vertebrate axis formation during embryonic development [41]. The protein encoded by *COBL* has been shown to belong to the family of WH2 (Wiskott–Aldrich Syndrome Protein Homology 2) repeat proteins that are expressed in a variety of morphogenetic and patterning processes and are involved in the regulation of actin assembly [42]. Recent single cell data from WAT samples of mice and humans suggest that *COBL* is primarily expressed in VAT mesothelial cells and is also detectable in SAT [43]. Furthermore, protein abundance of COBL in human SAT and VAT has been shown to be associated with the diabetic state of patients [44]. Interestingly, similar to the results obtained by us, two recent studies have identified *COBL* as differentially expressed in *UCP1*-expressing vs. *UCP1*-nonexpressing human AT samples from adult patients undergoing surgery in the thyroid region [29,30], supporting a potential relevance for BAT function. Although these studies suggested that COBL might be potentially involved in processes related to AT biology and metabolism, by now no study has addressed a relevance of COBL for (brown) adipocyte differentiation in vitro. We identified *COBL* as an upregulated gene in brown vs. white SAT depots of children. In contrast to this, *Cobl* is not differentially expressed in the AT depots of mice and is not affected by cold exposure, which is in line with previously published transcriptome data from murine white and brown adipocytes [29]. However, *Cobl* expression is increased during brown adipocyte differentiation in vitro indicating a potential role in processes related to BAT development. This assumption is supported by the findings that the inhibition of *Cobl* is associated with a decreased *Ucp1* expression levels in mature brown adipocytes in vitro. Furthermore, higher *COBL* expression in SAT is associated with a metabolically beneficial state in children, i.e., lower BMI SDS and decreased AT inflammation.

MKX is a homeodomain-containing protein, which has been initially identified as a transcription factor expressed in developing tendons in mice [32]. MKX has been extensively characterized as a key factor for the tenogenic differentiation of mesenchymal stem cells by regulating the expression of tendon-related genes [33]. It has been shown to be dynamically expressed in the developing mouse embryo including various mesoderm-derived tissues, e.g., the somites, the limb buds, the developing gonads and the kidneys [45]. Interestingly, in vitro analyses of tendon-derived stem cells isolated from 3-week-old rats revealed that MKX plays a role in the chondrogenic, osteogenic and adipogenic differentiation of these cells [31]. Furthermore, recent studies suggested that *MKX* is ubiquitously expressed in the tissues of adult humans, including SAT and VAT [43,46]. However, a regulatory function of *MKX* in AT and in white or brown adipocyte differentiation has not been investigated in detail so far and there is no data on a potential association of *MKX* with metabolic disease in humans. We identified *MKX* as one of the top upregulated genes in brown vs. white AT samples of children indicating a relevance for AT biology and metabolism. Using in vitro analyses, we further showed that similar to *Pparg*, a master regulator of adipogenesis [47], *Mkx* expression is increased during adipogenesis. Interestingly, *Mkx* expression has also been observed to increase during the tenogenic differentiation of bone marrow mesenchymal stem cells [33]. However, the inhibition of *Mkx* expression does not affect adipocyte differentiation per se, but is associated with decreased *Ucp1* expression in brown adipocytes. Similar to *COBL*, higher *MKX* expression in AT is linked to metabolically favorable parameters, e.g., lower BMI SDS, decreased AT inflammation and increased insulin sensitivity.

The most downregulated gene in UCP1^+^ compared to UCP1^−^ AT samples in our study was *MYOC*. Genetic variants in the *MYOC* gene have been linked to the formation of glaucoma, and previous research primarily focused on aspects related to this. *MYOC* expression has been detected in different tissues inside the eye, e.g., the trabecular meshwork [48], cornea, iris, ciliary body and retinal epithelium [49], but also in numerous human tissues outside the eye, e.g., skeletal muscle and heart [50]. *MYOC* expression has also been described in bone-marrow-derived mesenchymal stem cells of several species, with humans presenting the highest expression level. Studies in mice have provided evidence that *Myoc* is essential for the differentiation of these mesenchymal stem cells into osteoblasts in vitro and that *Myoc*-deficient mice are characterized by impaired osteogenesis in vivo [35]. Recent analyses of single cell data obtained from murine and human WAT samples further showed that *MYOC* is predominantly expressed in the stromal-vascular fraction of SAT [43]. However, a potential effect of the loss of MYOC on adipocyte formation has neither been addressed in those previous studies nor in other studies. Our results indicate that MYOC does not affect adipocyte differentiation in general but seems to play an inhibitory role in the regulation of *Ucp1* during brown adipocyte differentiation since the inhibition of *Myoc* resulted in enhanced *Ucp1* expression in brown adipocytes in vitro. In line with this *Myoc* expression was inhibited during white adipocyte browning in vitro, which was associated with increased *Ucp1* expression. This might potentially explain the downregulation of *MYOC* in the perirenal and subcutaneous UCP1^+^ vs. UCP1^−^ AT samples of our Leipzig AT Childhood cohort. When we analyzed a potential association of *MYOC* expression in SAT of children with obesity and related parameters in general, we did not observe an association with BMI SDS. However, we detected inverse relationships with *MYOC* expression with adipocyte size and serum leptin levels as parameters of AT dysfunction as well as with HOMA-IR indicating an association with early signs of metabolic disease.

Interestingly, results from several recent studies provided evidence for a relationship of presence and function of BAT with glucose metabolism and insulin sensitivity in children [17,18,51]. In contrast to our study, these analyses were focusing on BAT mass determined by magnetic resonance imaging [17] and basal BAT activity measured by infrared thermal imaging [18,51] in classical BAT depots in the supraclavicular region, while gene expression was not investigated in these previous studies. In this regard, it would be interesting to analyze whether the expression of *COBL*, *MKX* and *MYOC* as potential regulators of BAT development and function is associated with BAT mass or the activity of classical BAT depots.

One strength of our study is that we performed our study on the identification and characterization of potential regulators of BAT development and a potential association with childhood obesity and early signs of metabolic disease in children. Previous studies on the potential regulators of BAT biology were mainly performed in adults who often present more advanced stages of the disease, already suffer from obesity-related comorbidities, and have a longer exposure time to environmental compounds or drug treatments that might have an effect on AT biology. A second strength is that in addition to perirenal AT, we analyzed subcutaneous UCP^+^ and UCP^−^ AT depots, which might allow a more unbiased identification of the potential regulators of (brown) AT function due to the exclusion of depot-specific differences. However, due to ethical reasons and the fact that we obtained AT samples during routine surgeries of children, we did not have access to classical brown AT depots, e.g., in the neck region. It would be interesting to analyze differences in gene regulation in this classical brown AT depot in comparison to *UCP1*-expressing perirenal and subcutaneous AT samples.

Furthermore, several studies have provided evidence for a sexual dimorphism in BAT activation and WAT browning [52], and we cannot exclude an effect of gender on the regulation of *Cobl*, *Mkx* and *Myoc* expression during WAT browning in mice since we used only female mice for the experiments. However, since the vast majority of the studies have reported a higher susceptibility to WAT browning amongst females, female mice represent a suitable model for studying the underlying processes [52].

In conclusion, we identify *COBL*, *MKX* and *MYOC* as novel potential regulators of AT biology and function, and show that these genes are involved in the regulation of brown adipocyte formation in vitro. Furthermore, we provide evidence that the AT expression of *COBL*, *MKX* and *MYOC* is related to early signs of AT dysfunction and metabolic disease in children, indicating a potential role in the pathogenesis of these processes. In order to assess whether and how *COBL*, *MKX* and *MYOC* might represent putative and promising targets for therapeutic interventions promoting WAT to BAT conversion to reduce obesity, future studies should focus on the better understanding of the mechanisms by which they regulate brown adipocyte formation.

## 4. Material and Methods

### 4.1. Human AT Biopsies (Leipzig AT Childhood Cohort)

The AT samples analyzed in this study were part of the Leipzig AT Childhood cohort and were collected during elective surgery (i.e., abdominal surgery, back surgery, orthopedic surgery, herniotomy, orchidopexy) as previously described [24,53]. Exclusion criteria were genetic syndromes, malignant disease, generalized inflammation, diabetes, cardiovascular or peripheral artery disease. The study was approved by the local ethics committee of the University of Leipzig (Reg. No: 265-08, 265-08-ff; NCT02208141).

Anthropometric data were collected as previously described [54]. Height and body mass index (BMI) values were transformed into standard deviation scores (SDS) applying sex- and age-specific national reference data [55]. Measurements of serum parameters were performed by a certified laboratory (Institute of Laboratory Medicine, Clinical Chemistry and Molecular Diagnostics, University of Leipzig).

Adipocyte size was measured after the fixation of freshly isolated adipocytes in osmium tetroxide (Science Services, Munich, Germany) using a Coulter counter (Multisizer III; Beckmann Coulter, Krefeld, Germany). The number of AT macrophages was assessed by CD68 immunostaining (M0718, DAKO, Glostrup, Denmark) of paraffin-embedded AT sections using the DAKO REAL™ APAAP Immunocomplex system. Detailed protocols for the processing of AT samples of the Leipzig AT Childhood cohort have been previously described by Landgraf et al. [24].

The immunohistochemical analysis of UCP1 protein in AT samples was performed as previously described [25]. Briefly, AT samples were dehydrated, embedded in paraffin and 12 µm thick sections were prepared. Antigen unmasking was performed in Target Retrieval Solution (DAKO). Endogenous peroxidases were blocked by incubating AT sections in 3% hydrogen peroxide in Tris pH 7.56 for 10 min. AT sections were then incubated for 30 min with rabbit polyclonal anti-UCP1 antibody (1:500; ab10983, Abcam, Cambridge, UK) or without antibody as a negative control. The detection of UCP1 was performed using HRP-labelled goat anti-rabbit immunoglobulin (1:200, P0448, DAKO) and the Envision dual link system-HRP (DAKO).

The measurement of global gene expression in AT samples of selected probands was performed with Illumina HumanHT-12 v4 BeadChip arrays [26].

### 4.2. Cold Exposure Experiments in Mice

Female C57BL/6NTac mice were obtained from Taconic (Denmark) and subsequently housed under pathogen-free conditions (3–5 mice per cage) at 23 °C on a 12 h light/dark cycle in the local animal facility (MEZ—Medizinisch-Experimentelles Zentrum, University of Leipzig, Leipzig). Mice were fed a control chow diet (D12450J, 10 kJ% fat, high amylose starch, Ssniff, Ssniff^®^, Soest, Germany) and had ad libitum access to water and food. After two weeks of acclimatization, mice were single-housed for one week at thermoneutrality (30 °C) and thereafter housed at 30 °C or 8 °C for one week (n = 6 per group) [56]. After sacrificing, BAT, inguinal WAT (corresponding to subcutaneous AT (SAT)) and epigonadal WAT (corresponding to visceral AT (VAT)) were snap frozen in liquid nitrogen and stored at −80 °C until further use.

### 4.3. Cell Culture

Murine HIB1B brown preadipocytes were differentiated to mature brown adipocytes as previously described [57]. Briefly, HIB1B cells were maintained in Dulbecco’s modified Eagle’s medium (DMEM) with 10% fetal bovine serum and 1% penicillin/streptomycin at 5% CO_2_ and 37 °C. For differentiation, cells were cultured to confluence (day 0) and then exposed to differentiation medium 1 (0.5 mM IBMX, 0.25 µM dexamethasone, 20 nM insulin, 1 nM T3). After 48 h, cells were maintained in differentiation medium 2 containing 20 nM of insulin and 1 nM of T3. At five time points during brown adipocyte differentiation (days 0, 1, 3, 5, 7), cells were harvested for RNA isolation and on day 7 for Oil-Red O staining.

Murine 3T3-L1 cells were differentiated to mature white adipocytes as previously described [58]. Briefly, 3T3-L1 cells were maintained in Dulbecco’s modified Eagle’s medium (DMEM) with 10% fetal bovine serum and 1% penicillin/streptomycin at 5% CO_2_ and 37 °C. For differentiation, cells were seeded into 6-well plates (100,000 cells/well), cultured to confluence (day 0) and treated with differentiation medium containing 0.5 mM of IBMX, 1 µg/mL of insulin and 0.25 µM of dexamethasone. The culture medium was then changed on day 5, 7 and 10 to maintenance medium supplemented with 1 µg/mL of insulin. For the induction of white adipocyte browning, media were either supplemented with 2 µM of rosiglitazone or 8.3 nM of human BMP7 (R&D Systems, Minneapolis, MN, USA) during the whole period of adipocyte differentiation. Cells were harvested before adipogenic induction (day 0) and on days 4, 8 and 12 after adipogenic induction for RNA and protein isolation, and on day 12 for Oil-Red-O staining.

### 4.4. siRNA-Mediated Knockdown of Candidate Genes

HIB1B and 3T3L1 cells were transfected using the Neon Transfection System 100 µL Kit (Invitrogen, Carlsbad, CA, USA) as previously described [53]. Electroporation parameters were: pulse voltage of 1300 V, pulse width of 20 ms and pulse number 2. For electroporation, gene-specific ON-TARGETplus SMARTpool small interfering (si)RNAs (siCobl, siMyoc and siMkx) and ON-TARGETplus control reagent (Dharmacon, Lafayette, LA, USA) were used at a final concentration of 500 nM and a cell density of 6 × 10^6^ cells/mL.

After electroporation, cells were seeded in 6-well cell culture plates, cultured to confluence and differentiated as described before. The efficient knockdown of gene expression was confirmed on day 0 of adipocyte differentiation using quantitative real-time PCR.

### 4.5. RNA Isolation and Quantitative Real-Time PCR Analysis

Total RNA from HIB1B and 3T3-L1 cells was isolated using the RNeasy Mini Kit in combination with QIAshredder columns (Qiagen, Hilden, Germany) and reverse-transcribed into cDNA using M-MLV Reverse Transcriptase (Invitrogen, Darmstadt, Germany) and random hexamer [p(dN)_6_] primers (Promega, Madison, WI, USA). Quantitative real-time PCR was performed using the ABI 7500 or Quant Studio 3 Real-Time PCR Systems (Applied Biosystems, Darmstadt, Germany). Serial dilutions of purified PCR products or plasmid DNA of the respective target genes were used as standard curves on each plate. All samples were measured in triplicate and normalized to the mean of the two reference genes, beta-actin (*Actb*) and TATA-box binding protein (*Tbp*).

Total RNA isolation, cDNA synthesis and quantitative real-time PCR from subcutaneous AT samples from children of the Leipzig AT Childhood cohort were performed as previously described [59]. In order to minimize bias from variations in cDNA input levels or from potential inhomogeneity due to slight differences in source material normalization, the mean of the three reference genes, *ACTB*, *TBP* and hypoxanthine phosphoribosyltransferase 1 (*HPRT*), was used.

Analyses of gene expression in the AT of mice was performed in John Heiker’s lab according to established protocols [60,61,62]. RNA isolation was completed using the RNeasy Lipid Tissue Mini kit (Qiagen). Reverse transcription was performed using the QuantiTect Reverse Transcription Kit (Qiagen). Gene expression was quantified using the LightCycler System LC480 and LightCycler-DNA Master SYBR Green I Kit (Roche, Mannheim, Germany), as previously described [63]. Gene expression was normalized to the endogenous control gene *36b4* using the ΔΔCT method.

The primer and probe sequences used for quantitative real-time PCR are listed in Table S1.

### 4.6. Protein Isolation and Western Blot

Protein was isolated using the acetone precipitation method according to the protocol provided with the RNeasy Mini Kit (Qiagen). Briefly, frozen flow throughs from the RNA isolation process were diluted in ice-cold acetone and incubated over night at −20 °C. Afterwards, the tubes were centrifuged for 10 min at maximum speed and 4 °C in a benchtop centrifuge. The supernatant was then discarded, and the pellet air-dried. The pellet was washed in ice-cold ethanol and air-dried again before resuspension in RIPA buffer (150 mM NaCl, 0.1% Triton X-100, 0.5% sodium deoxycholate, 0.1% SDS and 50 mM Tris at pH8.0) supplemented with protease inhibitor (Complete Protease inhibitor cocktail, Roche).

Protein concentrations were determined using the Pierce^TM^ BCA Protein Assay Kit (Thermo Fisher Scientific, Waltham, MA, USA). A total of 10 µg of proteins were mixed with 4× Laemmli loading buffer (10% SDS, 1M Tris pH6.8, Glycerol, 1% bromophenol blue, 2M DTT), boiled at 99 °C for 5 min and separated by 12% SDS-PAGE followed by electrophoretic transfer to a polyvinylidene fluoride (PVDF) membrane. The membrane was blocked for 1 h at room temperature in Tris-buffered saline with Tween20 (TBS-T; 50 mM Tris pH7.5–8.0, 150 mM NaCl and 0.1% Tween 20) containing 5% non-fat milk. Afterwards the membrane was washed 3 × 5 min in TBS-T and incubated with anti-UCP1 (Abcam, ab10983, 1:1000) or anti-β-Actin (Abcam, ab8227, 1:1000) antibodies overnight at 4 °C. The next day the membrane was washed 3 × 5 min in TBS-T and then incubated with anti-rabbit secondary antibody (Cell Signaling, 7074S, 1:2000) for 1 h at room temperature. Pierce ECL+ Western Blotting Substrate (Thermo Fisher Scientific) was used for the detection of protein signals.

### 4.7. Oil-Red-O Staining

Oil-Red-O staining was performed on day 7 of the HIB1B adipocyte differentiation and day 8 or 12 of the 3T3-L1 differentiation. For this, a stock solution of 0.5 g Oil-Red-O/100 mL isopropanol was diluted to a ratio of 3:2 in Aqua dest. A total of 2 ml of Oil-Red-O working solution was used for each well of a 6-well plate. After incubation at 37 °C for 30 min, the plates were washed in distilled water and microscopic images were taken using an EVOS microscope from Fisher Scientific. Absorbance was determined at 540 nm using the FLUOstar OPTIMA (BMG LABTECH, Offenburg, Germany) after thorough washing with water and extraction of Oil-Red-O by incubation with isopropanol.

### 4.8. Statistical Analyses

Statistical analyses and the graphical presentation of results were performed using GraphPad Prism 6 (GraphPad Software, San Diego, CA, USA) and Statistica 13 (StatSoft, Tulsa, OK, USA). Data that did not adhere to a Gaussian distribution were log-transformed before analyses. Parametric tests (Pearson correlation analysis, two-sided Student’s *t*-test, and one-way ANOVA) were applied for quantitative traits and chi-squared tests applied for categorical traits. For group comparisons, patients with overweight and obesity were combined.

## Data Availability

The data analyzed in this study are available on request from the corresponding author. The data are not publicly available due to privacy reasons.

## Supplementary Materials

The following Supplementary Materials are available online at https://www.mdpi.com/article/10.3390/ijms24043085/s1.
