# Supplementary material for: COBL, MKX and MYOC Are Potential Regulators of Brown Adipose Tissue Development Associated with Obesity-Related Metabolic Dysfunction in Children
Source: Int J Mol Sci. 2023 Feb 4;24(4):3085. doi: 10.3390/ijms24043085 (PMC9964948; doi:10.3390/ijms24043085)
Supplement: Supplementary file 1 [file ijms-24-03085-s001.zip › Table S1.pdf]

Table S1. Primer and probe sequences for quantitative *real-time* PCR

Target Gene	Forward Primer	Reverse Primer	Probe
**Human: Analyses of adipose tissue samples**			
UCP1	ACGACACGGTCCAGGAGTTC	ACCAGCTAAAATCTTGCTTCCTAAAC	TCACCGCAGGGAAAGAAACAGCACC
COBL	AAGCAATAGTAAGGGCTGTTTAACG	CACGGACACCCCTGAGATG	CATCCATGCACTCACGTTCTCTTACGCTG
MKX	GTGTCTCCATCGTCATCAGAAACT	TTGCTTGGTCCTTTTCTGTTAGC	ATCGCACAGACACTCTGGAAAACGGATC
MYOC	AAGCGACTAAGGCAAGAAAATGA	CCAAATTCCACGTAGAAACTTCTCT	TGTCCCCAGACCCGAGACACTGC
ACTB	CGAGCGCGGCTACAGCTT	CCTTAATGTCACGCACGATTT	ACCACCACGGCCGAGCGG
TBP	TTGTAAACTTGACCTAAGACCATTGC	TTCGTGGCTCTCTTATCCTCATG	AACGCCGAATATAATCCCAAGCGGTTTG
HPRT	GGCAGTATAATCCAAAGATGGTCAA	GTCTGGCTTATATCCAACACTTCGT	CAAGCTTGCTGGTGAAAAGGACCCC
**Mouse: Analyses of adipose tissue samples**			
Pgc1a	CTTTTGTGGACGGAAGCAAT	GAGTCTTGGGAAAGGACACG	
Ucp1	CCGAAACTGTACAGCGGTCT	CCGAGAGAGGCAGGTGTTTC	
Cobl	TGTTACGCAGCCGTTCAAGT	GCAACAGTAAGGCTGAGCATCTT	TCGCCGGGTCCTCATCACTATCTGC
Mkx	TCAGAAACTGAAGGCACCTTTG	GCTTGGTCCCCTTCTGTTAGC	CTATCGCACAGACACCCCGGACATC
Myoc	Predesigned assay		(Mm00447900_m1, Life Technologies)
36b4	AAGCGCGTCCTGGCATTGTCT	CCGCAGGGGCAGCAGTGGT	
**Mouse: Analyses of HIB1B and 3T3-L1 cells**			
Pparg	Predesigned assay		(Mm01184322_m1, Life Technologies)
Ucp1	Predesigned assay		(Mm01244861_m1, Life Technologies)
Cobl	TGTTACGCAGCCGTTCAAGT	GCAACAGTAAGGCTGAGCATCTT	TCGCCGGGTCCTCATCACTATCTGC
Mkx	TCAGAAACTGAAGGCACCTTTG	GCTTGGTCCCCTTCTGTTAGC	CTATCGCACAGACACCCCGGACATC
Myoc	Predesigned assay		(Mm00447900_m1, Life Technologies)
Actb	GCTCTGGCTCCTAGCACCAT	GCCACCGATCCACACCGCGT	TCAAGATCATTGCTCCTCCTGAGCGC
Tbp	AATCTTGGCTGTAAACTTGACCTAAAG	CGTGGCTCTCTTATTCTCATGATG	TCGTGCAAGAAATGCTGAATATAATCCCAAGC

Primers and probes are given in 5´-3´direction.
