# Supplementary material for: COBL, MKX and MYOC Are Potential Regulators of Brown Adipose Tissue Development Associated with Obesity-Related Metabolic Dysfunction in Children
Source: Int J Mol Sci. 2023 Feb 4;24(4):3085. doi: 10.3390/ijms24043085 (PMC9964948; doi:10.3390/ijms24043085)
Supplement: Supplementary file 1 [file ijms-24-03085-s001.zip › Table S2.pdf]

Table S2. Patient characteristics for samples with Illumina transcriptome profiles

	UCP1^{+}			UCP1^{-}		
	n	Mean±SEM	Range	n	Mean±SEM	Range
**Perirenal AT samples**						
Male/Female (% male)		3/3 (50)			1/1 (50)	
Age [years]	6	1.6±0.7	0.4–4.7	2	9.1±8.8	0.3–18.0
PH	6	1 ±0	1	2	3 ±2	1–5
BMI SDS	5	-0.5 ±0.7	-2.1–1.8	2	0.1±0.5	-0.4 –0.5
**Subcutaneous AT samples**						
Male/Female (% male)		4/0 (100)			4/0 (100)	
Age [years]	4	2.9±1.7	0.3–7.9	4	4.6±1.8	1.1–7.9
PH	4	1 ±0	1	4	1 ±0	1
BMI SDS	4	-1.3 ±0.2	-1.9 – -0.8	4	-0.8 ±0.6	-1.8 –0.8

Data are grouped into UCP1^{+} and UCP1^{-} AT samples according to histological analyses. UCP1^{+}, positive for uncoupling protein 1; UCP1^{-}, negative for uncoupling protein 1; AT, adipose tissue; PH, pubertal stage; BMI, body-mass index; SDS, standard deviation score
