# Supplementary material for: COBL, MKX and MYOC Are Potential Regulators of Brown Adipose Tissue Development Associated with Obesity-Related Metabolic Dysfunction in Children
Source: Int J Mol Sci. 2023 Feb 4;24(4):3085. doi: 10.3390/ijms24043085 (PMC9964948; doi:10.3390/ijms24043085)
Supplement: Supplementary file 1 [file ijms-24-03085-s001.zip › Table S4.pdf]

Table S4. Cohort characteristics of probands and samples included in quantitative real-time PCR analysis

	Lean			Overweight and Obese			
	**N**	**Mean±SEM**	**Range**	**N**	**Mean±SEM**	**Range**	**p**
Male/Female (% male)	171	108/63 (63.2)	-	101	57/44 (56.4)	-	0.273[a]
Age [years]	171	7.6±0.4	0.1–18.4	101	11.9±0.4	1.0–18.4	**<0.001**
PH	137	2.1±0.1	1–6	91	3.0 ±0.2	1–6	**<0.001**
BMI SDS	168	-0.2±0.1	-3.2–1.2	101	2.2±0.1	1.3–4.3	**<0.001**
Adipocyte size [µm]	28	112.1±2.6	83.2–134.5	40	123.2±2.3	98.0–146.2	**0.002**
Macrophages per 100 adipocytes	141	7.0±0.6	0–29	87	13.7±1.8	0–115	**<0.001[b]**
Leptin [ng/ml]	89	5.8±0.6	0.2–28.2	87	28.8±2.5	0.6–99.0	**<0.001[b]**
HOMA-IR	118	1.3±0.1	0.1–5.6	87	3.3±0.3	0.1–12.7	**<0.001[b]**
COBL expression [A.U.]	170	199±21	6–2,193	101	184±48	5–4,766	**0.029[b]**
MKX expression [A.U.]	171	355±38	9–4,060	100	152±17	10–1,138	**<0.001[b]**
MYOC expression [A.U.]	171	21,695±1,801	143–141,979	100	18,739±2,211	33–199,029	0.876[b]

[a]For sex, statistical significance was determined by chi-squared test. [b]Statistical analyses were performed for log-transformed parameters. Significant *p*-values are indicated in bold. PH, pubertal stage according to pubic hair (PH); BMI SDS, body mass index standard deviation score; HOMA-IR, homeostasis model assessment of insulin resistance; COBL, cordon-bleu WH2 repeat protein; MKX, mohawk homeobox; MYOC, myocilin
